# Crohn’s Disease and Axial Spondyloarthritis: From Systemic Inflammation to Amyloidosis

**DOI:** 10.3390/jcm15114188

**Published:** 2026-05-28

**Authors:** Daria Alexeevna Kutsakina, Alexandra Dmitrievna Chernichkina, Nadezhda Andreevna Nikolaeva, Olga Olegovna Voronkova, Olga Valerevna Tashchyan, Marina Genrikovna Mnatsakanyan, Yuri Nikitich Belenkov, Sergey Viktorovich Osminin, Fedor Petrovich Vetshev, Ildar Ravilievich Bilyalov, Alexander Sergeevich Panferov

**Affiliations:** 1 Hospital Therapy No. 1, Sechenov First Moscow State Medical University, Moscow 119991, Russia; chernichkina_a_d@staff.sechenov.ru (A.D.C.); nikolaeva_n_a@staff.sechenov.ru (N.A.N.); voronkova_o_o@staff.sechenov.ru (O.O.V.); tashchyan_o_v@staff.sechenov.ru (O.V.T.); mnatsakanyan_m_g@staff.sechenov.ru (M.G.M.); belenkov_yu_n@staff.sechenov.ru (Y.N.B.); 2 Faculty Surgery No. 1, Sechenov First Moscow State Medical University, Moscow 119991, Russia; osminin_s_v@staff.sechenov.ru (S.V.O.); vetshev_f_p@staff.sechenov.ru (F.P.V.); bilyalov_i_r@staff.sechenov.ru (I.R.B.); 3 Faculty Therapy No. 1, Sechenov First Moscow State Medical University, Moscow 119991, Russia; panferov_a_s@staff.sechenov.ru

**Keywords:** clinical report, Crohn’s disease, axial spondyloarthritis, AA amyloidosis, biologic therapy

## Abstract

**Background:** Crohn‘s disease (CD) is frequently complicated by extraintestinal manifestations, including axial spondyloarthritis (axSpA). Both diseases share genetic (HLA-B27, IL23R, ERAP1/2) and immunopathological mechanisms (Th17/IL-23 axis). Their co-occurrence increases the risk of systemic complications such as AA amyloidosis. **Case presentation:** We report a 42-year-old male with HLA-B27-positive axSpA who developed CD shortly after initiating secukinumab (IL-17A inhibitor). Following discontinuation of secukinumab and surgical management of CD, the patient experienced rapidly progressive AA amyloidosis affecting the kidneys and intestines, leading to acute kidney injury and requiring hemodialysis. Potential triggering factors included a preceding intestinal infection and self-administered infrared physiotherapy. **Conclusions:** Coexistent CD and axSpA significantly increases the risk of severe AA amyloidosis. IL-17 inhibitors should be used with extreme caution in patients with subclinical or active CD. Early screenings for proteinuria and low-threshold biopsy are essential to detect AA amyloidosis. In patients with both conditions, TNF-α or IL-12/23 inhibitors are preferred over IL-17 blockade.

## 1. Introduction

Inflammatory bowel diseases, in particular Crohn’s disease (CD), represent a significant medical and social problem in modern gastroenterology. This is primarily due to their increasing incidence among young adults of working age, with disease manifestation occurring before the age of 30 [1,2]. It should be noted, however, that citation [1] reflects Russian epidemiological data, while citation [2] provides global estimates from the Global Burden of Disease study; figures vary substantially by region and are influenced by economic development and diagnostic practices. The presence of extraintestinal manifestations further aggravates the disease course. Epidemiological data indicate that up to half of all patients with CD experience at least one extraintestinal manifestation [3]. In some patients, these symptoms become the first signs of the underlying disease even before the onset of characteristic intestinal symptoms. One of the extraintestinal manifestations in patients with CD is axial spondyloarthritis (axSpA), which occurs in approximately 5–15% of patients [4]. However, some studies using modern imaging techniques report an even higher prevalence, reaching up to 20–25% [5,6]. Axial spondyloarthritis encompasses both radiographic (ankylosing spondylitis) and non-radiographic forms, with the latter diagnosed by magnetic resonance imaging before structural changes become visible on X-rays [7]. Despite different organ localizations (intestine in CD and axial skeleton in axSpA), both diseases share common etiopathogenetic mechanisms. The most striking example is the association with the HLA-B27 antigen, which is detected in 90–95% of patients with axSpA compared to 7–8% in the general population [8]. The main mechanisms by which HLA-B27 influences disease pathogenesis include the “arthritogenic peptide” hypothesis and the abnormal presentation of autoantigens to CD8+ T lymphocytes [9]. A key role is also played by the tendency of the HLA-B27 molecule to misfold in the endoplasmic reticulum, which triggers cellular stress responses and excessive synthesis of proinflammatory cytokines. This misfolding propensity has been directly demonstrated in cellular models and is thought to drive the unfolded protein response (UPR), contributing to enhanced IL-23 expression [10,11]. An additional factor is the formation of HLA-B27 homodimers on the cell membrane, which can directly activate NK cells and Th17 cells, perpetuating the vicious cycle of autoinflammation [9]. Although the frequency of HLA-B27 is not increased in isolated CD, this marker is found significantly more often in patients with combined pathology (CD + axSpA) [12]. In addition to HLA-B27, both diseases are associated with polymorphisms in the IL23R, ERAP1, and ERAP2 genes, indicating shared molecular mechanisms underlying their development [11]. The central link in the pathogenesis of both diseases is dysregulation of the immune response with a predominance of Th17-mediated inflammation. Interleukin-23 (IL-23), produced by activated dendritic cells and macrophages, stimulates the differentiation and proliferation of Th17 lymphocytes [13]. These cells, in turn, secrete the proinflammatory cytokines IL-17 and IL-22, which in axSpA cause entheseal inflammation and subsequent ankylosis [14], while in CD they lead to intestinal epithelial damage and transmural inflammation [15]. In this context, the use of secukinumab—a monoclonal antibody that selectively inhibits IL-17A—is pathogenetically justified; it is currently officially approved for the treatment of axSpA [16]. Furthermore, the combination of these two nosologies in a single patient increases the risk of systemic complications, including amyloidosis [17]. Amyloidosis is characterized by extracellular deposition of insoluble fibrillary proteins, leading to progressive dysfunction of target organs. In CD and axSpA, secondary (AA) amyloidosis predominantly develops, affecting the kidneys, gastrointestinal tract, and other organs. Its diagnosis is often delayed due to the nonspecific nature of clinical presentations. We describe a clinical case of concurrent CD and axSpA that was complicated by rapidly progressive AA amyloidosis of the kidneys and intestine, resulting in acute kidney injury and requiring hemodialysis. Of particular interest is the difficulty in identifying the triggering factor and determining its impact on the severity of the attack: a preceding intestinal infection, the use of infrared physiotherapy procedures during treatment of axSpA with secukinumab (which is used with caution in CD).

## 2. Clinical Case Description

Patient K., a 42-year-old male, was admitted to the gastroenterology department of University Clinical Hospital No. 1, Sechenov University, with complaints of abdominal pain upon changes in body position that progressively worsened over 4 days, mushy stools up to 3 times per day, flatulence, abdominal distension, increased sweating, and decreased urine output.

History taking revealed that 2 months earlier, the patient had experienced episodes of diarrhea with mushy stools up to 6 times per day and a fever up to 38 °C. He received outpatient antibiotic therapy (amoxicillin + clavulanic acid). However, after 2 weeks, his condition deteriorated: vomiting up to 3 times per day, loose stools increased to 7–10 times per day, bright red blood on toilet paper, and a fever up to 39 °C. The patient was hospitalized in an infectious diseases hospital with a diagnosis of “unspecified gastroenteritis and colitis of infectious origin, moderate severity.” Infectious etiology was ruled out during workup, and the patient was discharged with only marginal improvement (mushy stools persisted).

It was also known that at the age of 20, the patient was diagnosed with HLA-B27-positive axial spondyloarthritis (bilateral sacroiliitis grade IV, bilateral coxitis, symphysitis, enthesopathies), for which biologic disease-modifying antirheumatic therapy (secukinumab 150 mg subcutaneously, 5 injections) was initiated. However, after 5 injections, this treatment was discontinued due to the onset of episodes of mushy stools.

Worsening of symptoms was noted after self-administration of infrared radiation to the abdominal area 4 days prior.

Physical examination: the abdomen was distended and not participating in respiration. Palpation revealed abdominal wall tension and tenderness in all quadrants, with positive peritoneal signs. Body temperature—38 °C; heart rate—98 beats/min; respiratory rate—20 breaths/min; and SpO_2_ on room air—93%. Blood tests revealed mild normochromic normocytic anemia (red blood cells 4.1 × 10^12^/L, hemoglobin 101 g/L) and elevated inflammatory markers (ESR 36 mm/h, CRP 298.7 mg/L, presepsin 236 pg/mL, procalcitonin 16.74 ng/mL). Additionally, protein metabolism disorder syndrome (total protein 59 g/L, albumin 19.6 g/L) and electrolyte disturbance syndrome (potassium 3 mmol/L, sodium 125.6 mmol/L) were noted. Computed tomography of the chest and abdomen revealed bilateral small hydrothorax and pneumoperitoneum (Figure 1).

The patient was transferred to the surgical department, where suturing of multiple colonic defects, resection of the cecal dome, and creation of a double-barrel ileostomy were performed (Figure 2, Figure 3 and Figure 4).

Histological examination confirmed Crohn’s disease involving the small and large intestine, including the following findings: transmural granulomatous inflammation with formation of deep ulcerative defects and fistulous tracts, areas of pyloric metaplasia, lipomatosis, and fibrosis of the submucosal layer (Figure 5 and Figure 6).

The postoperative period was uneventful. The patient received antibiotic therapy (meropenem—1.0 g three times daily intravenously, polymyxin B—50 mg twice daily intravenously). Correction of nutritional deficiency, fluid-electrolyte balance, and acid-base status were also performed.

One month after surgery, a fever up to 38 °C and abdominal pain developed. Blood tests revealed anemia (hemoglobin 88 g/L), inflammatory syndrome (CRP 170.6 mg/L), and signs of protein metabolism disorder (total protein 55 g/L, albumin 20.6 g/L). Blood and urine cultures grew Klebsiella pneumoniae, which was interpreted as sepsis. Antibiotic therapy (meropenem—1.0 g three times daily intravenously, vancomycin—240 mg four times daily via ileostomy) and red blood cell transfusions were administered, with clinical and laboratory improvement. Two weeks later, pathogenetic therapy for CD was initiated (prednisolone—5 mg twice daily via ileostomy, hydrocortisone—100 mg twice daily as rectal suppositories). Remission of CD was achieved on this regimen, after which elective ileostomy closure was performed.

However, one month later, the patient developed complaints of severe weakness and loss of appetite. Laboratory findings showed nephrotic syndrome (proteinuria, decreased total protein and albumin, dyslipidemia) with impaired nitrogen excretory function (creatinine increased to 600 μmol/L and elevated urea, estimated GFR 18 mL/min by CKD-EPI). Based on these clinical and laboratory findings, amyloidosis was suspected. A colon biopsy confirmed AA amyloidosis with involvement of the intestine and kidneys. A trial of colchicine at a dose of 0.25 mg daily was attempted; however, the patient developed nausea, vomiting of ingested food, and weakness. Renal failure continued to progress, prompting transfer to the “Artificial Kidney” unit for initiation of renal replacement therapy in the form of hemodialysis.

Notable findings included impaired consciousness, signs of secondary infection, severe hypoalbuminemia, electrolyte disturbances, and cachexia, all attributable to active inflammatory bowel disease. Following comprehensive therapy (hemodialysis, antibacterial and antifungal agents, vasopressor support, glucocorticosteroids, transfusions of fresh frozen plasma and washed red blood cells, anticoagulants, electrolyte correction, gastroprotective therapy), positive dynamics were achieved. Laboratory findings showed a decrease in creatinine (from 726 μmol/L to 372 μmol/L) and urea (from 28 mmol/L to 10 mmol/L), a decrease in CRP (from 168.8 mg/L to 58 mg/L), and an increase in hemoglobin (from 88 g/L to 103 g/L). The patient was discharged for outpatient follow-up by an internist, nephrologist, rheumatologist, and gastroenterologist, with a recommendation to initiate disease-modifying therapy for the underlying diseases using biologics and cytostatics.

Final diagnosis: Crohn’s disease involving the small and large intestine, fistulizing phenotype, with minimal activity (4 points on the Harvey–Bradshaw index). Prerenal acute kidney injury of mixed (hypovolemic, urate) etiology, resolving to stage 4 chronic kidney disease (CKD-4). Axial spondyloarthritis, HLA-B27-positive, treated with biologic therapy (secukinumab). Bilateral sacroiliitis, grade IV by Kellgren, bilateral coxitis (more pronounced on the left), osteonecrosis of the subchondral region of the left femoral head, symphysitis, enthesopathies.

Complications: malabsorption syndrome, with hypoalbuminemia and sideropenia; moderate mixed-mechanism anemia (iron deficiency, anemia of chronic disease); severe protein–energy malnutrition; cachexia; severe nutritional deficiency; electrolyte disturbances; AA amyloidosis with gastrointestinal and renal involvement; systemic inflammatory response syndrome (Klebsiella pneumoniae cultured from blood); acute cardiovascular insufficiency; and disseminated intravascular coagulation (DIC).

## 3. Discussion

The described clinical case illustrates a severe course of Crohn’s disease in a patient with axial spondyloarthritis, complicated by the development of AA amyloidosis of the intestine and kidneys, progressing to end-stage renal failure requiring hemodialysis. Today, CD and axSpA are no longer viewed as completely independent conditions. On the contrary, their combination reflects shared immunoinflammatory mechanisms. Both diseases are associated with dysregulation of the immune response, impaired intestinal barrier function, and activation of proinflammatory cytokines [18,19].

At the same time, this case clearly demonstrates that a shared inflammatory cascade does not guarantee equivalent efficacy of the same targeted therapy. The onset of intestinal symptoms in this patient chronologically coincided with the initiation of secukinumab therapy for axial spondyloarthritis. Secukinumab is a fully human monoclonal antibody that neutralizes IL-17A, a cytokine implicated in both entheseal inflammation in axSpA and intestinal barrier regulation. This circumstance deserves particular attention because secukinumab, an IL-17A inhibitor, has been associated in several studies with exacerbation of CD [20]. This is likely because IL-17 in the intestinal mucosa participates not only in sustaining inflammation but also in maintaining epithelial barrier integrity [21]. Therefore, IL-17 blockade, although justified in axSpA, may have unfavorable consequences in a patient with a predisposition to inflammatory bowel disease. Admittedly, in the present observation, it cannot be unequivocally stated that secukinumab was the cause of CD onset. Nevertheless, the temporal sequence itself is clinically relevant and merits discussion, as it underscores the need for vigilance regarding intestinal manifestations in patients receiving IL-17 inhibitors. This is particularly important when, prior to therapy initiation, the patient had already experienced episodes of loose stools, abdominal pain, or other symptoms that could have been early manifestations of inflammatory bowel disease. From a practical standpoint, this case raises the question of therapy selection in patients with coexisting CD and axSpA. In such a situation, preference should likely be given to those treatment options capable of controlling both diseases simultaneously without increasing the risk of IBD exacerbation. Instead of IL-17 inhibitors, IL-12/23 inhibitors and TNF-alpha inhibitors, which block higher stages of the inflammatory cascade, should be used for the treatment of CD [16,21].

The presented observation also highlights the importance of timely diagnosis and monitoring of systemic complications in patients with chronic inflammatory diseases, especially when they coexist. In this patient, prolonged and insufficiently controlled systemic inflammation led to the development of one of the most severe complications—AA amyloidosis. Amyloidosis in this case became a key factor determining the patient’s poor prognosis, underscoring the need for early aggressive anti-inflammatory and immunosuppressive therapy to suppress underlying disease activity. The development of acute kidney injury and the need for renal replacement therapy required a multidisciplinary approach involving a gastroenterologist, nephrologist, rheumatologist, and surgeon.

A literature search (PubMed, 2000–2025) identified only six previously reported cases of AA amyloidosis secondary to coexistent CD and axSpA. In most, amyloidosis developed after more than a decade of active disease. Our case is unique due to the rapid progression (within months of CD diagnosis) and the temporal association with secukinumab. This raises the hypothesis that IL-17 blockade might accelerate fibrotic and amyloidogenic pathways in susceptible individuals, though this remains speculative. Regarding infrared physiotherapy, no published evidence supports its role as a trigger of IBD flares; therefore, we cannot attribute causality, but we report it as a potential confounder.

## 4. Conclusions

This case provides three key lessons for clinical practice:

1. Screening before IL-17 inhibition: Patients with axSpA should be evaluated for subclinical intestinal inflammation (e.g., fecal calprotectin, ileocolonoscopy if symptoms present) before initiating secukinumab or other IL-17 blockers. Otherwise, undiagnosed CD may flare, leading to severe complications.
2. Preferred biologics for coexistent CD and axSpA: TNF-α inhibitors (infliximab, adalimumab) or IL-12/23 inhibitors (ustekinumab) are safer and effective choices, whereas IL-17 inhibitors are best avoided.
3. Amyloidosis surveillance: In patients with long-standing, poorly controlled CD and axSpA, annual assessment for proteinuria and low-threshold biopsy of affected organs are essential to detect AA amyloidosis early, before irreversible organ failure develops.

The selection of therapy in such patients requires extreme caution and a personalized approach aimed at rapid, complete suppression of systemic inflammation to prevent irreversible complications.

## Figures and Tables

**Figure 1:**
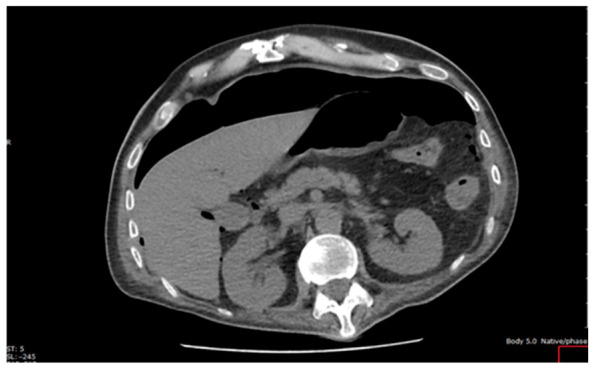
CT of the chest and abdominal cavity: no fresh lesions or infiltrative changes in the lungs; bilateral small hydrothorax; pneumoperitoneum (source is likely located in the dome of the cecum/in the region of the gastric antrum); signs of colitis; and microliths in both kidneys.

**Figure 2:**
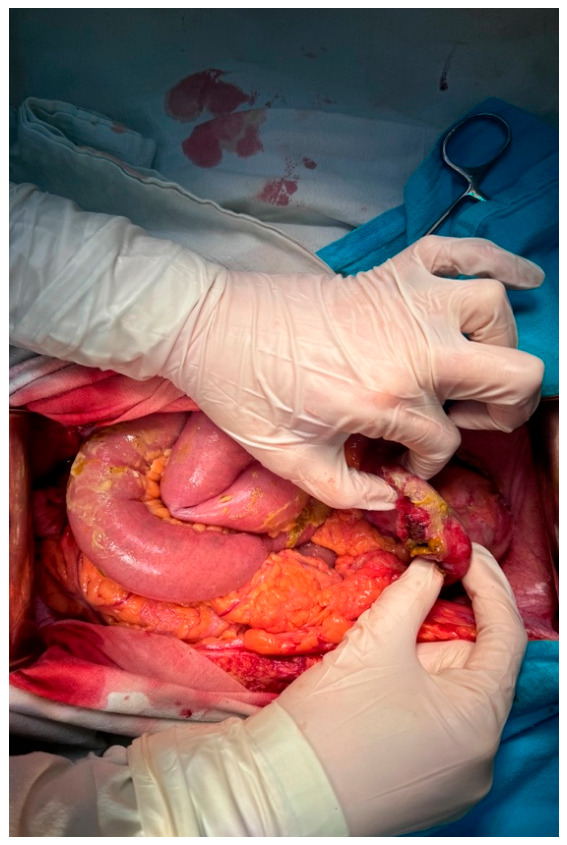
Intraoperative findings: distended loops of the small bowel, peristalsis is absent. In the region of the cecal dome, a defect was noted.

**Figure 3:**
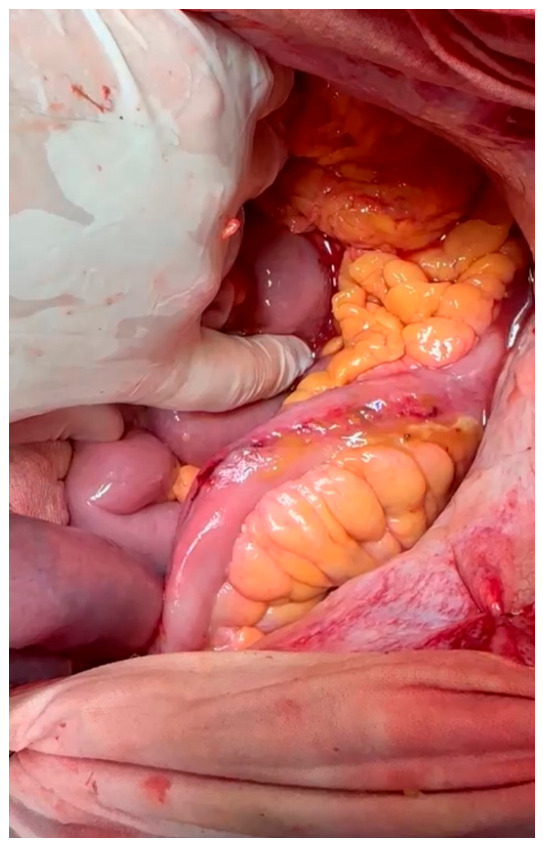
Intraoperative findings: in the transverse colon, two defects were identified.

**Figure 4:**
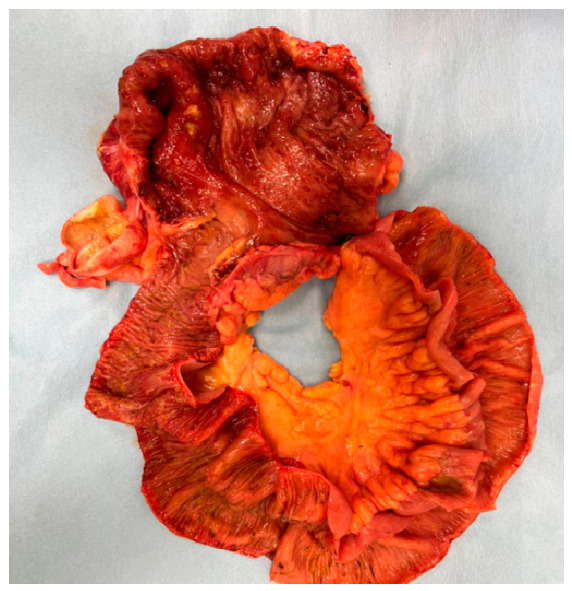
Resected affected fragment of the large intestine.

**Figure 5:**
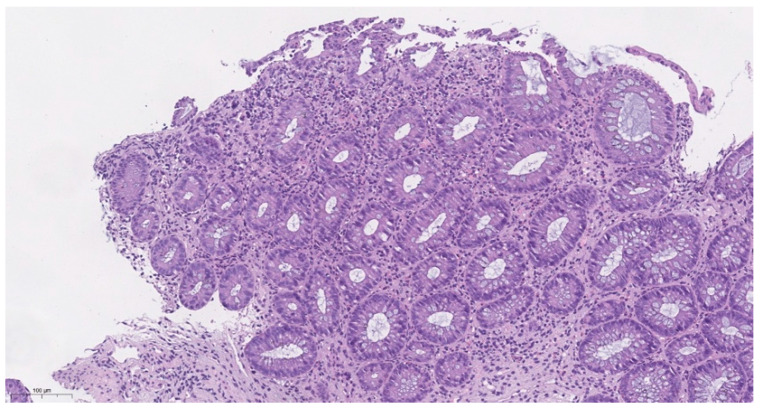
Histopathological findings in a rectal biopsy specimen of chronic active erosive proctitis. The biopsy specimen shows an eroded surface, crypt deformation, hyperplasia with a decrease in the number of goblet cells, and inflammatory infiltration in the lamina propria. Hematoxylin and eosin (H&E) stain; mag. 200×.

**Figure 6:**
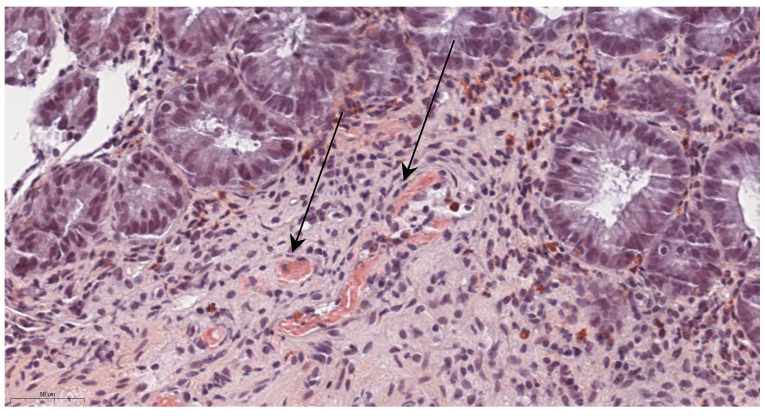
Microspecimen of a vessel: staining with Congo red yielded positive red staining of amyloid deposits in the walls of small vessels (indicated by arrows). Congo red stain; mag. 250×.

## Data Availability

The original contributions presented in this study are included in the article. Further inquiries can be directed to the corresponding author.

## Abbreviations

The following abbreviations are used in this manuscript:

AA	Secondary amyloidosis
axSpA	Axial spondyloarthritis
CD	Crohn’s disease
CKD	Chronic kidney disease
CRP	C reactive protein
DIC	Disseminated intravascular coagulation
eGFR	Estimated glomerular filtration rate
ESR	Erythrocyte sedimentation rate
HLA B27	Human leukocyte antigen B27
IL	Interleukin
IL-17A	Interleukin-17A
IL-23	Interleukin-23
NK	Natural killer cell
SpO_2_	Peripheral capillary oxygen saturation
Th17	T helper 17 cell
TNF-α	Tumor necrosis factor alpha
